# Performance Optimization of Polymer Fibre Actuators for Soft Robotics

**DOI:** 10.3390/polym12020454

**Published:** 2020-02-14

**Authors:** Ivan D. Rukhlenko, Syamak Farajikhah, Charles Lilley, Andre Georgis, Maryanne Large, Simon Fleming

**Affiliations:** Institute of Photonics and Optical Science (IPOS), School of Physics, The University of Sydney, Camperdown, NSW 2006, Australia

**Keywords:** pneumatic actuators, polymer fibres, soft robotics, Young’s modulus, drawing soft polymers

## Abstract

Analytical modeling of soft pneumatic actuators constitutes a powerful tool for the systematic design and characterization of these key components of soft robotics. Here, we maximize the quasi-static bending angle of a soft pneumatic actuator by optimizing its cross-section for a fixed positive pressure inside it. We begin by formulating a general theoretical framework for the analytical calculation of the bending angle of pneumatic actuators with arbitrary cross-sections, which is then applied to an actuator made of a circular polymer tube and an asymmetric patch in the shape of a hollow-cylinder sector on its outer surface. It is shown that the maximal bending angle of this actuator can be achieved using a wide range of patches with different optimal dimensions and approximately the same cross-sectional area, which decreases with pressure. We also calculate the optimal dimensions of thin and small patches in thin pneumatic actuators. Our analytical results lead to clear design guidelines, which may prove useful for engineering and optimization of the key components of soft robotics with superior features.

## 1. Introduction

Recent decades have seen explosive growth in the robotics industry [1,2,3]. The implementation of robots into domestic and medical contexts requires addressing the potential for conventional rigid designs to harm human operators or bystanders. This problem can be substantially mitigated with the use of soft robotics, since softer materials and compliant designs are inherently more forgiving on impact [4,5,6,7,8,9]. Soft robotics is also the most promising prospect if we want robots in the home in the near future [10,11,12].

The rise of the 3D printer and drops in the cost of traditional manufacturing methods have led to significant expansion in the soft-robotics industry [13,14,15,16,17,18,19,20]. The commercial space has rapidly adopted pneumatic claws where there is a demand for delicate handling of goods, like the handling of fruits and delicate parcels. So called ‘bi-bellows’ designs of pneumatic actuators dominate this field [21,22].

However, soft robotics in its current evolution faces a number of challenges [23,24,25]. First and foremost, soft robots are generally expensive to manufacture, and even more expensive to maintain if they become faulty. This is a consequence both of the cost of appropriate polymer stocks and of the specialized machinery required to print parts. Cheap conventional poly-carbonate plastics are generally unable to withstand the repeated motion and accompanying stresses in actuating mechanisms, so specialized polymers are often required. The geometric complexity of pneumatic designs usually makes 3D printers the only viable manufacturing technology. Further, while there exist commercial high-precision 3D printers (with nanometer scale resolution), their high cost and slow speed makes them inappropriate for a mass-production model. A new paradigm shift to soft robotics is required to make such technologies cheaper and suitable for large scale production.

Scaling down conventional rigid actuators comes with three major issues: (i) as a design is scaled down, the cost of production and manufacture rises rapidly; (ii) the high rigidity of actuating mechanisms is not ideal in many environments; and (iii) delivering substantial force from smaller mechanisms is a significant engineering challenge.

The third issue has been partially solved with the advent of small-scale robots [26,27,28,29,30,31,32,33], which are however ‘small’ only where the robot actually interfaces while the rest of the design is as large as, if not larger than, a conventionally sized mechanism. As yet there is no solution to the first issue of price, and the benefits of such innovations as robotic surgery are not likely to reach the developing world for many years. Only the second issue of rigidity is currently being addressed by the soft-robotics paradigm [34,35,36,37,38]. A paradigm shift like the one aforementioned has the potential to minimize the first issue of price with ease.

In summary, soft robots today are too expensive and lack a general fabrication platform, however, recent advances in drawing soft polymer fibres may provide solutions to these problems. It has been shown that soft polymers such as polyurethane can be thermally drawn down to extremely narrow tubes (fibers) at the scale of a few hundred microns [39,40,41]. More importantly, the process of mass producing these polymer tubes is far cheaper than those used for producing other soft robotic designs such as the bi-bellows actuator [42,43].

This low cost of production and the ability for significant size variation can address the issues previously discussed, making soft polymer tubes potentially superior candidates to existing soft robotics technologies. Using such materials as the sole constituent of a soft robotic actuator requires the creation of new pneumatic designs, since existing models like bi-bellows are only possible with the design flexibility offered by the relative precision of 3D printers. A brief review of existing technologies makes it evident that asymmetry in design is required to achieve pneumatic actuation.

In this paper, we present a systematic theoretical study to optimize the cross-section of soft polymer tubes which may serve as a new material base for soft pneumatic actuators. We specifically maximize the bending angle of a soft pneumatic actuator realized as a circular polymer tube with an asymmetric patch in the shape of a hollow-cylinder sector attached to its outer surface. The general expression for the actuator’s bending angle is analyzed to calculate the optimal parameters of the patch and formulate useful design guidelines that can facilitate engineering and optimisation of the key components of soft robotics.

## 2. Results

### 2.1. Statics of Pneumatic Actuators

**Figure 1:**
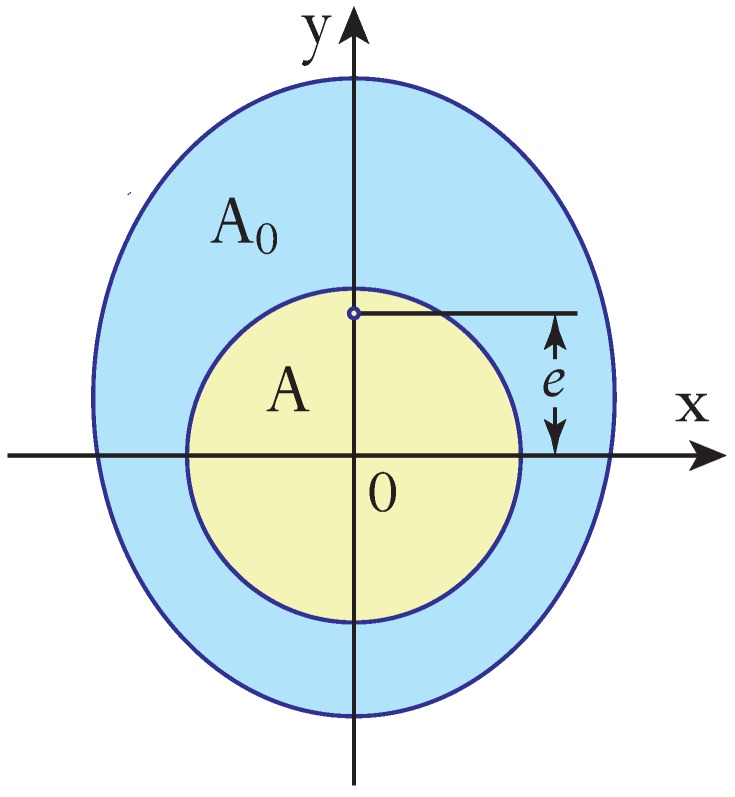
Cross-section of pneumatic actuator showing position of its neutral axis (small open circle) and illustrating parameters *e*, *A*, and $A_{0}$. Shaded in blue and yellow are the polymer tube and the pressurized hollow core of the actuator.

In the design of pneumatic actuators, engineers are usually interested not only in maximizing the forces produced in response to a given pressure but also in maximizing the deflections [44]. To calculate the latter, we shall begin by considering a generic pneumatic actuator made of a hollow polymer tube of Young’s modulus *E*, length *L*, and cross-sectional area $A_{0}$. If the neutral axis of the tube does not coincide with the centroid of the cross-section, which is the centre of pressure, then pressurization of the tube causes it to elongate and bend in the direction of the neutral axis. Without loss of generality we take the unpressured actuator to be parallel to the *z* axis and have a plane of symmetry x=0. Then the neutral axis lies in the yz plane, as shown in Figure 1, and deflection occurs in the same plane.

The elongation δL of the actuator is caused by the tensile force F=PA, which is the product of the applied pressure *P* and the cross-sectional area *A* of the pressurized volume of the tube, $\delta L=LF/(A_{0}E)$. The pure bending of the unloaded actuator is caused by the bending moment M=eF, which is directly proportional to the offset *e* between the neutral axis and the centre of pressure. In the adopted geometry this offset is equal to the *y* coordinate of the neutral axis and given by [45]

(1) $$e=\frac{1}{A_{0}}\int y\;dA,$$

where the integral is evaluated over the entire cross-sectional area $A_{0}$.

In what follows, we assume that the actuator is not loaded in the transverse direction, and thus the magnitude of the bending moment is constant over its entire length. In this case the deflection curve representing the shape of the actuator’s axis after bending is a part of a circle of radius r=EI/M, which is determined by the area moment of inertia *I* with respect to the neutral axis. According to the parallel axis theorem, the moment *I* is expressed through the second moment of inertia $I_{0}$ with respect to the centroid as

(2) $$I=I_{0}+A_{0}e^{2},$$

where

(3) $$I_{0}=\int y^{2}\;dA.$$

Using the above notations and denoting $\varepsilon =eA_{0}$, we obtain the bending angle ϑ=(L+δL)/r of the actuator as [46]

(4) $$\vartheta \left(P\right)=L\left(1+\frac{P}{E}\frac{A}{A_{0}}\right)\frac{P}{E}\frac{A}{A_{0}}\frac{\varepsilon }{I},$$

where the linear first term in the parenthesis (∝P) comes from pure bending and the nonlinear second term $\left(\propto P^{2}\right)$ is due to elongation. One can see that pure bending gives the main contribution to ϑ(P) at relatively low pressures, $P\ll (A/A_{0})E$, whereas elongation dominates when the pressure is high, $P\gg (A/A_{0})E$.

Equation (4) is applicable when both the axial stress $\left(\sigma_{z}\right)$ and the circumferential stress $\left(\sigma_{\varphi }\right)$ inside the tube do not exceed the ultimate tensile strength of the polymer. For bending to be elastic, these stresses must also be less than the yield stress $\sigma_{0}$. If the internal pressure satisfying this condition is still high enough to alter the cross-section of the actuator, coefficients $A/A_{0}$ and ε/I become functions of *P*.

### 2.2. Bending of Hollow-Tube Pneumatic Actuator

**Figure 2:**
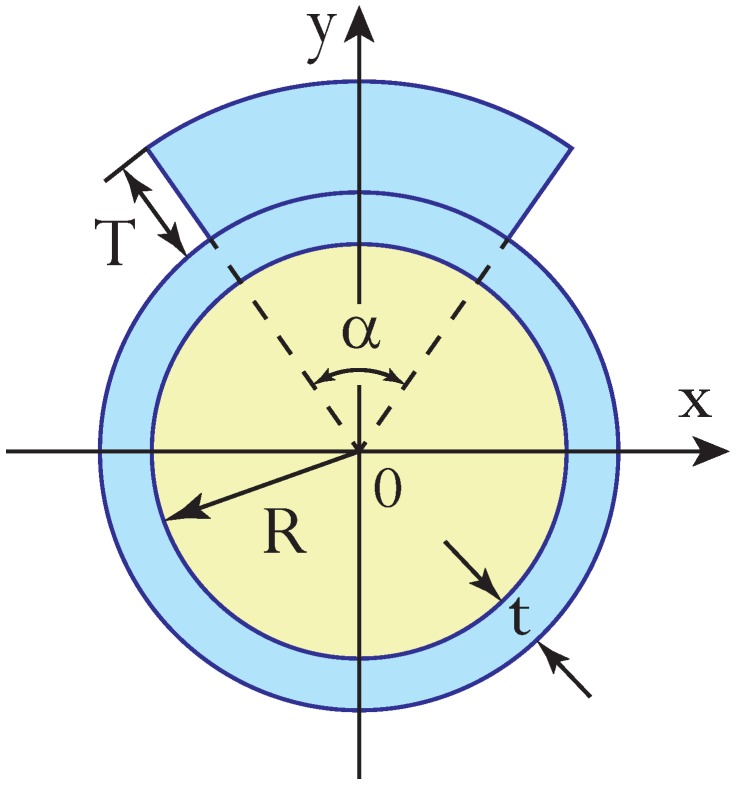
Cross-section of pneumatic actuator made of hollow polymer tube of radius *R* and thickness *t* and polymer patch of angular width α and thickness *T* on top of it.

We now apply the above formalism to the pneumatic actuator whose cross-section is shown in Figure 2. The actuator is composed of a polymer tube of radius *R* and wall thickness *t* and a small polymer patch of angular width α and thickness *T* made of the same material. This geometry is chosen due to the ease of its fabrication with the standard fiber drawing technique and will be considered throughout the rest of the paper.

In the particular case of the cross-section shown in Figure 2, we have

(5) $$\begin{array}{c} A=\pi R^{2}, \end{array}$$

(6) $$\begin{array}{c} A_{0}=\pi t^{2}q\left(\alpha ,\tau \right), \end{array}$$

(7) $$\begin{array}{c} \varepsilon =2\sin\left(\alpha /2\right)t^{3}f_{3}\left(\tau \right), \end{array}$$

(8) $$\begin{array}{c} I_{0}=\left[\left(1/2\right)\left(\alpha +\sin\alpha \right)f_{4}\left(\tau \right)-\pi f_{4}\left(-1\right)\right]t^{4}, \end{array}$$

where

(9) $$\begin{array}{c} q\left(\alpha ,\tau \right)=2\rho +1+\frac{\alpha }{2\pi }\left(2\rho +2+\tau \right)\tau , \end{array}$$

(10) $$\begin{array}{c} f_{n}\left(\tau \right)=\frac{{\left(\rho +1+\tau \right)}^{n}-{\left(\rho +1\right)}^{n}}{n}, \end{array}$$

and we have introduced the relative patch thickness τ=T/t and the relative tube radius ρ=R/t.

The maximal pressure inside the actuator can be estimated from the requirement of its integrity as follows. In steady state, the force generated by the internal pressure must be balanced by the force produced by the axial stress applied to the actuator cross-section in the xy plane, which yields $\sigma_{z}=\left(A/A_{0}\right)P$, as well as by the circumferential stress applied to its cross-section in the xz plane, which gives $\sigma_{\varphi }=\rho P$. Since both stresses should not exceed the yield stress of the polymer, the internal pressure is limited by the condition

(11) $$\begin{array}{c} P≲\min\left\{\sigma_{0}\left(A_{0}/A\right),\sigma_{0}/\rho \right\}=\sigma_{0}/\rho . \end{array}$$

Substituting Equations (5)–(10) in Equation (4), the following equation for determining the bending angle of the hollow-tube pneumatic actuator is obtained:

(12) $$\vartheta \left(\alpha ,\tau \right)=\kappa \rho^{3}\frac{L}{R}\left(1+\frac{\kappa \rho^{2}}{q(\alpha ,\tau )}\right)\frac{2\sin\left(\alpha /2\right)f_{3}\left(\tau \right)}{p(\alpha ,\tau )},$$

in which κ=P/E is the relative pressure factor and

(13) $$p\left(\alpha ,\tau \right)=\left[\left(1/2\right)\left(\alpha +\sin\alpha \right)f_{4}\left(\tau \right)-\pi f_{4}\left(-1\right)\right]q\left(\alpha ,\tau \right)+\left(4/\pi \right)\sin^{2}\left(\alpha /2\right)f_{3}^{2}\left(\tau \right).$$

It should be noted that this equation is valid for pressures that are generally much lower than those determined by Equation (11), because it assumes that the cross-section of the actuator remains unchanged.

As expected, the bending angle vanishes for α=0, α=2π, and τ=0—due to the axial symmetry of the actuator, as well as for τ=∞—due to the infinite flexural rigidity of the patch,

(14) $$\begin{array}{c} \vartheta (0,\tau )=\vartheta (2\pi ,\tau )=0, \end{array}$$

(15) $$\begin{array}{c} \vartheta (\alpha ,0)=\vartheta (\alpha ,\infty )=0. \end{array}$$

Consequently, there are optimal widths and thicknesses of the patches (α and T) that maximize the bending angle of the pneumatic actuator for a given pressure factor (κ) and fixed geometric parameters of the tube (L, *R*, and t).

From general physical considerations, it is clear that conditions similar to Equations (14) and (15) exist for actuators of other cross-sections, which means that their bending performance can also be optimized by tuning the geometric parameters of the asymmetric features.

## 3. Discussion

It is instructive to begin analysing the general expression for the bending angle by considering *thin actuators* made of relatively thin tube and patch (t≪R and T≪R) and characterized by parameters ρ≫1 and τ≪ρ. In this case $f_{n}\left(\tau \right)\approx \rho^{n-1}\tau$ and Equations (12) and (13) are simplified to the form

(16) $$\vartheta \left(\alpha ,\tau \right)=\mu \frac{L}{R}\left(1+\frac{\mu /2}{1+(\alpha /2\pi )\tau }\right)\frac{\pi \tau \sin(\alpha /2)}{\chi (\alpha ,\tau )},$$

where μ=κρ and

(17) $$\chi \left(\alpha ,\tau \right)=\pi^{2}\left(1+\frac{\alpha }{2\pi }\tau \right)\left(1+\frac{\alpha +\sin\alpha }{2\pi }\tau \right)+2\tau^{2}\sin^{2}\left(\alpha /2\right).$$

The bending performance of thin actuators is seen to be controlled by a single pressure-dependent structural parameter μ, which is the product of the pressure factor and the relative tube radius. From Equation (11) the values of μ are estimated to be limited by the ratio of yield stress to Young’s modulus, $\mu ≲\sigma_{0}/E$. This ratio depends on the type of polymer (see Table 1), ranging from about 0.28 for silicone elastomers (SE) to 17 for polyurethane elastomers (elPU) [47]. From Equation (16) it can be concluded that elongation of pneumatic actuators has little effect on their bending if they are made of polymers with $\sigma_{0}≲2E$ but may become crucial at high pressures for polymers with $\sigma_{0}\gg 2E$.

It is easy to verify that Equations (14) and (15) are still satisfied, implying that the bending angle of thin actuator has a maximum as a function of both its arguments.

### 3.1. Tubes of Equal Thicknesses

A thin pneumatic actuator can be fabricated by co-drawing a thin hollow tube of circular cross-section with a part of a similar tube serving as the patch. In this case τ=1 and the bending angle is a function of α only. The optimal patch thickness, determined by the condition $\vartheta_{\alpha }^{\prime }\left(\alpha ,1\right)=0$, can be calculated from the transcendental equation

(18) $$\frac{\mu }{4\pi }s\left(\alpha \right)=\left(1+\frac{\alpha }{2\pi }\tau \right)\left(\frac{\mu }{2}+1+\frac{\alpha }{2\pi }\tau \right)s^{\prime }\left(\alpha \right),$$

where s(α)=sin(α/2)/χ(α,1). For μ=0 and μ≫1 the roots of this equation are given by

(19) $$\begin{array}{c} \alpha_{0}\approx 0.83\;\pi \approx 149{}^{\circ}, \end{array}$$

(20) $$\begin{array}{c} \alpha_{\infty }\approx 0.62\;\pi \approx 112{}^{\circ}. \end{array}$$

**Figure 3:**
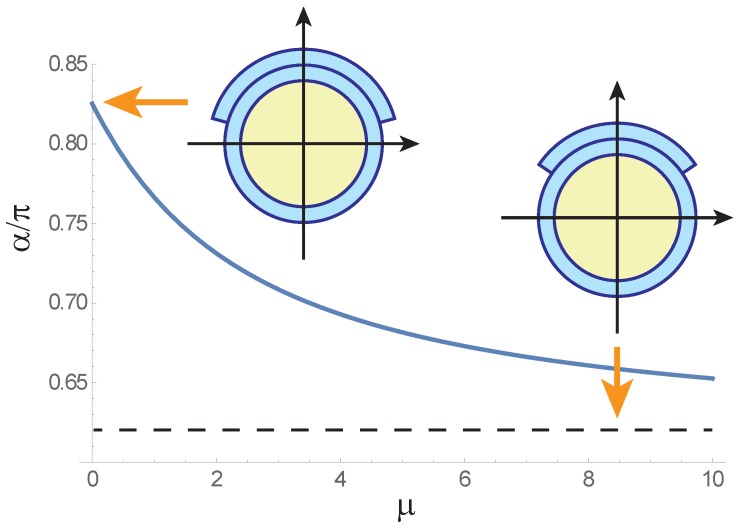
Optimal patch width of thin pneumatic actuator as function of pressure-dependent structural parameter μ=(P/E)(R/t). The thicknesses of the patch and the tube are alike. Insets show optimal actuator cross-sections for μ=0 and μ≫1.

Figure 3 shows the optimal patch width plotted as function of μ. The monotonic decay of this function is accompanied by the growth of the maximal bending angle according to Equation (16). The growth is linear at low pressures when μ≲0.1 and pure bending dominates, $\vartheta_{\max}\approx 0.133\;\left(L/R\right)\mu$, and quadratic for larger μ, $\vartheta_{\max}\approx 0.066\;\left(L/R\right)\mu^{2}$. The insets in the figure show cross-sections of actuators corresponding to the optimal angles $\alpha_{0}$ and $\alpha_{\infty }$.

### 3.2. A Small Asymmetric Patch

Another actuator that can be easily analyzed is the one where the patch is so narrow that sinα≈α. Then the equation for calculating ϑ will be

(21) $$\vartheta \left(u\right)=\mu \frac{L}{R}\left(1+\frac{\mu /2}{1+u}\right)\frac{u}{1+3u+4u^{2}},$$

where u=τα/(2π) and hence the bending angle is fully determined by the product of the relative patch thickness and width.

The optimal *u* obeys the equation

(22) $$\mu \left(8u^{3}+7u^{2}-1\right)=2\left(1+u^{2}\right)\left(1-4u^{2}\right)$$

whose roots for μ=0 and μ≫1 are

(23) $$\begin{array}{c} u_{0}=1/2\;\mathrm{and}\;u_{\infty }\approx 0.323. \end{array}$$

**Figure 4:**
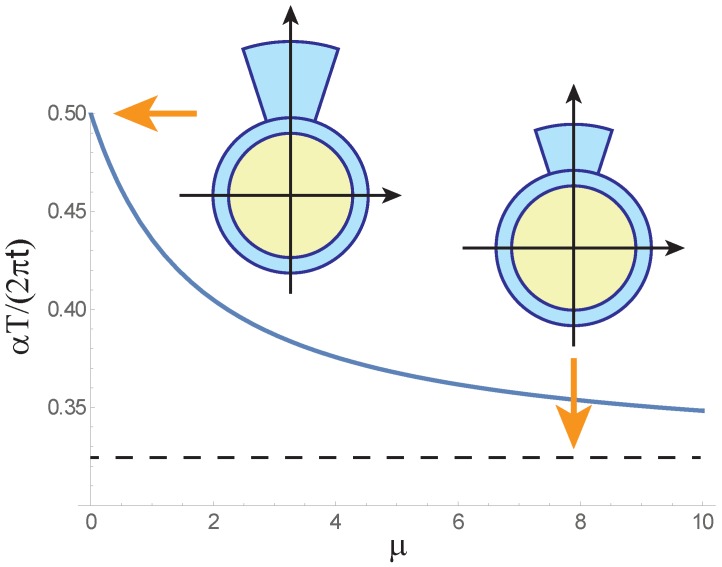
Optimal parameter u=αT/(2πt) of thin pneumatic actuator as function of pressure dependent structural parameter μ=(P/E)(R/t). Insets show optimal cross-sections of actuator with α=π/5 for μ=0 and μ≫1.

The optimal *u* is plotted as a function of μ in Figure 4. Similar to the previous case, the decay of this function leads to the linear growth of the maximal bending angle for μ<0.1 when the actuator elongation is negligible, $\vartheta_{m}\approx 0.143\;\left(L/R\right)\mu$, and to its quadratic growth for larger μ, $\vartheta_{m}\approx 0.071\;\left(L/R\right)\mu^{2}$. Cross-sections shown in the inset of the figure correspond to the optimal parameters $u_{0}$ and $u_{\infty }$.

### 3.3. Optimization in General Case

The preceding discussion was for the case of relatively thin actuators. In the instance in which either *t* or *T* (or both) is comparable to *R*, it is not permissible to use Equation (16) and we must recourse to the exact Equation (12). This is evidenced by the fact that the bending angle of thin actuators does not have an absolute maximum but peaks over a hyperbola-shape ridge τα=const [c.f. Equations (16) and (21)]. On the other hand, the bending angle given by the exact expression has a single maximum, which determines the optimal values of α and τ as functions of κ and ρ for any parameters of the actuator.

**Figure 5:**
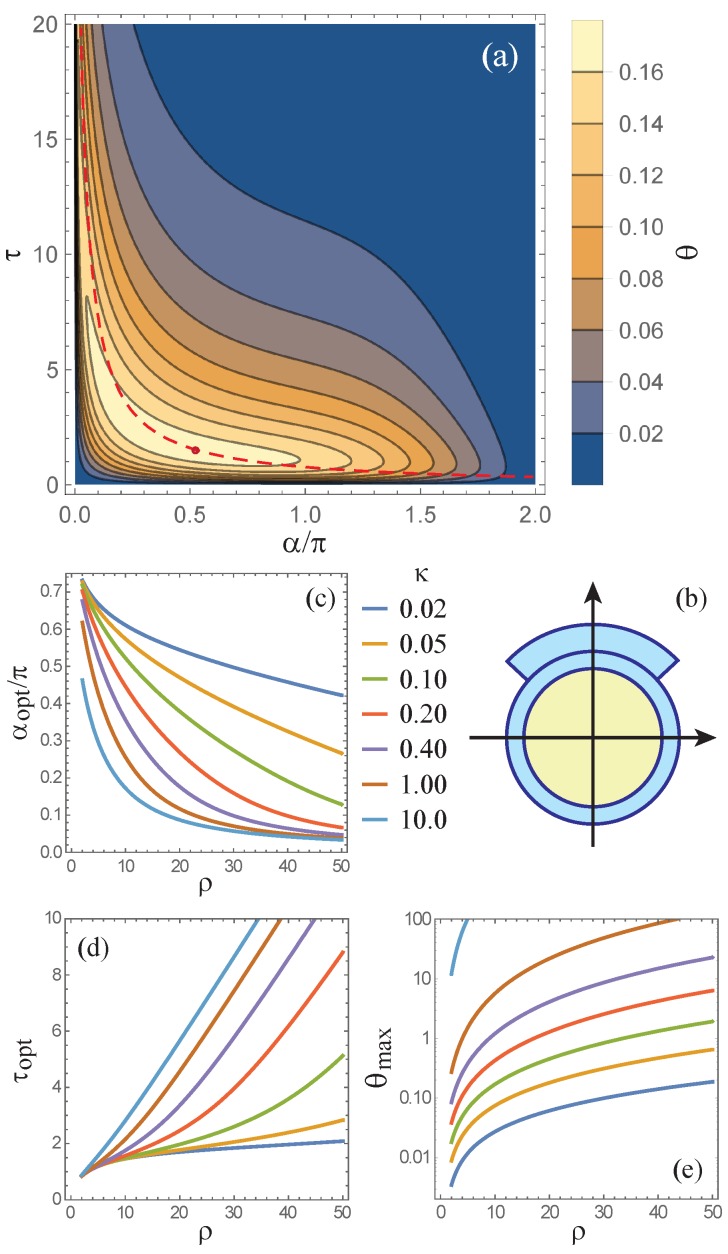
(**a**) Contour plot of function ϑ(α,τ) [Equation (12)] for κ=0.1 and ρ=10; (**b**) optimal actuator cross-section (with $\alpha_{\mathrm{opt}}\approx 0.52\approx 94{}^{\circ}$ and $\tau_{\mathrm{opt}}\approx 1.49$) corresponding to the maximal bending angle shown by the open circle in (**a**); and (**c**–**e**) optimal α and τ and the maximal bending angle $\vartheta_{\max}=\vartheta \left(\alpha_{\mathrm{opt}},\tau_{\mathrm{opt}}\right)$ as functions of ρ for different κ. In all cases it was assumed that R=L.

The normalized bending angle of the pneumatic actuator [Equation (12) with R=L] is plotted in Figure 5. In agreement with the limiting behavior expressed by Equations (14) and (15), the bending of the actuator is the strongest for one set of optimal parameters $\left(\alpha_{\mathrm{opt}},\tau_{\mathrm{opt}}\right)$ corresponding to the peak of function ϑ(α,τ) in Figure 5a. The cross-section defined by the optimal actuator’s parameters is shown in Figure 5b. Owing to the finite width of the peak and its ridge-like shape, it is possible to use a narrower patch at the expense of increasing its thickness without significantly reducing the bending angle. The approximate tradeoff between the two optimal parameters can be estimated from Figure 4 and for μ=1 is given by $\alpha_{\mathrm{opt}}\tau_{\mathrm{opt}}\approx 2\pi \times 0.44$. This tradeoff is shown by the dashed hyperbola in Figure 5a.

Figure 5c,d show the optimal width and thickness of the asymmetric patch as functions of relative tube radius R/t for different relative pressures P/E. One can see that the optimal width monotonically decreases with R/t and that the higher the pressure applied to the actuator, the steeper the decrease. This trend is opposite to the monotonic growth of the optimal thickness, which becomes less and less steep with the buildup of pressure. The functional dependencies of the optimal dimensions of the patch on R/t suggest that bending of thinner tubes requires higher and higher asymmetry of the actuator’s cross-section as the pressure grows bigger. This conclusion is rather general and holds for actuators of other cross-sectional shapes.

The maximal bending angle $\vartheta_{\max}$ achievable with the optimal dimensions $\alpha_{\mathrm{opt}}$ and $\tau_{\mathrm{opt}}$ of the pneumatic actuator is shown in Figure 5e. One can see that the optimized actuator can yield very high bending angles for even relatively low pressures. For example, in agreement with the colour scale of the contour plot, for ρ=10 and κ=0.1 we have $\vartheta_{\max}\approx 0.17$. This value corresponds to a 360°-bending of actuators with L≈37R and P=E/10. It should be noted that since the pressure applied to the actuator is limited by the maximal circumferential stress it can withstand before breaking or significantly changing its cross-section, the maximal bending angle can be calculated from Figure 5e only for $\rho ≲(\sigma_{0}/E)/\kappa$ [see Equation (11)]. Hence, the higher the yield stress of the polymer, the larger the bending that can be achieved with this polymer for a given actuation pressure.

Summarizing the above results, we can formulate the following general conclusions and design guidelines for soft pneumatic actuators made of hollow polymer tubes. First, the bending angle of soft pneumatic actuators scales linearly with the ratio of their length to the inner tube radius and can be maximized for a fixed pressure by tuning the cross-section of the asymmetric patch. Second, there is a tradeoff between the width and thickness of the optimal asymmetric patch allowing one to achieve almost the maximal bending angle using a wide range of patches. Third, the bending angle of thin pneumatic actuators (with t≪R and T≪R) is determined by the pressure-dependent parameter μ=(P/E)(R/t), which should not exceed the ratio of yield stress to Young’s modulus *E* of the polymer. Fourth, if the thicknesses of the patch and the main hollow tube of the actuator are alike, the optimal angular width of the patch varies between 149° at low pressures, P≲(t/R)(E/10), and 112° at high pressures, P≫(t/R)(E/10). Fifth, the product $\left[\alpha_{\mathrm{opt}}/\left(2\pi \right)\right]\left(T_{\mathrm{opt}}/t\right)$ of the optimal relative dimensions of the narrow patch of thin pneumatic actuators is a pressure-dependent constant, which varies from 1/2 at low pressures to about 0.323 at high pressures.

## 4. Conclusions

We have optimized the cross-section of a soft pneumatic actuator to achieve its maximal bending for a fixed actuation pressure. By applying a general analytical model of pneumatic actuators with arbitrary cross-sections to an actuator realized as a circular polymer tube with an asymmetric patch in the shape of a hollow-cylinder sector, it was shown that the strongest deflection of the actuator is achievable with different optimal dimensions of the patch, which depend on the applied pressure. We also calculated the optimal patch dimensions for thin actuators and formulated general design guidelines for soft pneumatic actuators in terms of their relative material and geometric parameters. We believe that our results will benefit the development of soft robotics and facilitate the design of new integral components of soft robotic systems.

## Figures and Tables

**Table 1:** Characteristic values of Young’s modulus *E*, yield stress $\sigma_{0}$, and their ratio for different polymers (from Ref. [47]).

Polymer	*E* (MPa)	$\sigma_{0}$ (MPa)	$\sigma_{0}/E$
Silicone elastomers	5–20	2.4–5.5	0.28–0.48
Ethylene-vinyl acetate	10–40	12–18	0.5–1.2
Butyl rubber	1–2	2–3	1.5–2
Neoprene	0.7–2	3.4–24	4.9–12
Natural rubber	1.5–2.5	20–30	12–13.3
Isoprene	1.4–4	20–25	6.3–14.3
Polyurethane elastomers	2–3	25–51	12.5–17

## Abbreviations

The following key notations are used in this manuscript:

*E*	Young’s modulus
$\sigma_{0}$	yield stress
*P*	pressure inside the actuator
*e*	distance from the neutral axis to the centroid
*I*	area moment of inertia with respect to the neutral axis
$I_{0}$	area moment of inertia with respect to the centroid
$A_{0}$	cross-sectional area of the polymer tube
*A*	cross-sectional area of the pressurized volume of the polymer tube
*L*	length of the polymer tube
δL	elongation of the polymer tube
*R*	internal radius of the polymer tube
*t*	thickness of the polymer tube
α	angular width of the polymer patch
*T*	thickness of the polymer patch
ϑ	bending angle of the actuator
